# Report of Three Cases of Abscess of the Brain

**Published:** 1882-09

**Authors:** J. T. Eskridge

**Affiliations:** Physician to the Howard and St. Mary’s Hospitals


### Selections.

EXPLANATION.

In the August number of the JOURNAL AND EXAMINER we published an interesting selected article on "Micrococci in Malignant Diseases," by Dr. JOHN M. KEATING, which was forwarded to us in proof-sheets, without any information in regard to the journal office from which the proofs came. We have since learned that they belonged to the *Medical Times*, of Philadelphia. The following articles, by J. T. ESKRIDGE, M.D., and WILLIAM BARTON HOPKINS, M.D., have been received in the same manner, and evidently from the same office. We consequently credit them to the *Medical Times*, of Philadelphia.—ED.

---

REPORT OF THREE CASES OF ABSCESS OF THE BRAIN. By J. T. ESKRIDGE, M.D., Physician to the Howard and St. Mary's Hospitals. Read May 3, 1882.

Abscess of the brain is of sufficiently frequent occurrence to make the subject of interest to the general practitioner. The cases reported in this paper are all the more interesting because each, due to a different cause, was associated with dissimilar diseases.

The first case was associated with cancer in several of the other organs of the body; the second, with chronic meningitis; the third, with apparent facial erysipelas and pneumonia, followed by acute meningitis.

Detailed cases of brain lesions lose half of their interest, and e come almost valueless to students of cerebral localization, when unattended by accurate descriptions of the relations of pathological lesions to ganglia, fissures, and convolutions.

I wish to acknowledge the services of my friend Dr. Charles K. Mills, who took an active part in the post-mortem examination of Case I., and rendered me valuable assistance in accurately locating the lesions in the first two cases.

CASE I. *Two Abscesses of the Brain.*—1. The larger one, involving the centrum ovale of the postero-frontal and parietal lobes, deeper portions of the gray matter of the anterior parietal convolution, and superior parietal lobule; 2. The smaller, the anterior two-thirds of the lenticular nucleus, and the adjoining portions of the external capsule and claustrum.

September 8, 1881, Mrs. D., æt. 54, born in Germany of a family several of whose members had died from cancer, had been a sufferer from headache fourteen years. She had a large frame and carried considerable fat. Excepting the head trouble, she had enjoyed excellent health until two years before, when she noticed a small tumor involving the left mammary gland. This proved to be cancerous, grew gradually and was removed by a surgeon one year after its first appearance. There had been no return of the growth, although some pain had been complained of in the region from which it had been excised.

Her head pain had greatly increased in severity during the last few months.

In the early part of July, 1881, the left arm began to feel weak, and ten or twelve days later, the left leg was similarly affected, but to a less extent. About this time she went to the country for her health. Continuing to grow worse, she returned home during August, three weeks before I first saw her, and was able to walk from the street cars to her house, a few hundred yards distant. She was then treated by a homœopathic physician for a few weeks, when I was called in, and made the following notes:

The left leg was almost completely paralyzed, the muscles feeling soft and flabby. The left arm was entirely paralyzed. The muscles of the left side of the face were slightly paretic. The right pupil was normal in size, left slightly dilated, and responded imperfectly to light. There was no paralysis of the muscles of the tongue, palate, or pharynx. The tongue was slowly protruded in the median line, but when it was held in this position a few seconds it became tremulous, a vibratory tremor taking place in individual muscular fibers. Muscular power was impaired in the right arm and leg, but co ordination was perfect. She had had no convulsions, and there was no muscular twitching.

With the exception of a slight tendency to drooping of the upper lid of the left eye, the ocular muscles seemed to be acting well and in harmony.

Sensation was unimpaired on both sides of the body.

*Special Senses.*—Vision was equal in each eye, and she read Snellen No. 8, held at about fourteen inches. The ophthalmoscope revealed nothing beyond a slight haziness of the right retina. Taste, smell, and hearing were nearly normal, being well preserved for a woman of her age. Speech was unaffected.

The tongue was moist and slightly coated, appetite poor, bowels constipated, not having been opened for several days. The urine was high colored, sp. gr. 1025, but free from albumen. The bladder was tolerant and acted voluntarily.

She was restless, did not sleep well, and complained bitterly of pain in her head. The pain was usually dull. Its seats of intensity were at the top of the head and above each temple, being worse on the right side. She was intelligent, her mind was clear, but being under the impression that her cancer would return, she worried, had become emotional and imaginative.

Temperature (left axillary) 99° ; pulse 76, full, soft, and regular ; respirations 24 per minute.

September 10, two days later, during my absence from the city, she was seen for me by Dr. Mills, who reported her to be about in the same condition.

September 12. She was not so intelligent, worried more, and refused to eat. The feet were extended and inverted, the balls of the great toes lying in contact with each other. The internal and posterior muscles of the legs were so firmly contracted that it required considerable force on the part of the attendant to evert and flex the feet, and the effort gave her decided pain. The muscles of the left side of the face were almost completely paralyzed. Swallowing solid or semi-solid substances was so difficult that she refused to eat.

Temperature, right axilla 96°; left axilla 99.4°; right elbow 95°; left elbow 96.7°. Pulse, 86; respirations 26 per minute. Respiration was largely of the abdominal type. After taking citrate of magnesium, her bowels were opened once yesterday, being the first time in about a week.

September 13. At 11 a.m., the temperature of the room being 80°, after ascertaining her body heat, as indicated in each axilla, I made observations on her cerebral temperature, applying the thermometer to most of the stations used by Broca, Gray, and later by Mills. On the present occasion, the surface temperature of the head was taken at eight different stations, on a subsequent one, at two, and still later at eleven. Hick's surface thermometer was employed and allowed to remain in placc ten minutcs. On each occasion the temperature of the room was ascertained, and that of each axilla noted. The observations were made by myself between the hours of 11 a.m. and 2 p.m. The selected stations were as follows: 1. A middle frontal station in the center of the forehead. 2. A right anterior frontal station, over the right eye, an inch and a half above the brow, and the same distance from the median line of the forehead. 3. A left anterior frontal station, similarly located on the left side. 4. A right posterior frontal station, near the external angular process of the frontal bone. 5. A left posterior frontal station, corresponding in position on the left side to that on the right. 6. A right parietal station, just above the right ear. 7. A left parietal station, just above the left ear. 8. A right Rolandic station, situated on or near a line drawn over the top of the head from the right to the left auditory meatus, and from two to three inches above the top of the right ear. 9. A left Rolandic station situated similarly to that on the right. 10. A right occipital station, one and a half inches to the right of the center of the occipital region. 11. A left occipital station, one and a half inches to the left of the center of the occipital region.

I shall now give the temperature records in connection with the history of the case, and further details under the head of remarks.

Temperature in right axilla, 97.9°; in left axilla, 98.4°. Pulse 104, weak and unsteady; respirations, 24 per minute.

*Surface Temperature of the Head.*

| RIGHT SIDE. | | | | LEFT SIDE. | | | |
|---|---|---|---|---|---|---|---|
| Anterior frontal station, | | | 97.3° | Anterior frontal station, | | | 97.7° |
| Posterior | " | " | 97.8° | Posterior | " | " | 98.1° |
| Parietal | " | " | 97.1° | Parietal | | " | 97.9° |
| Rolandic | " | " | 96.3° | Rolandic | | " | 96.8° |

She was unable to void her urine for the first time to-day. Thirty-two ounces were drawn off by the catheter. From this time to the date of her death, just two months later, the catheter was daily required to relieve the bladder. She had not eaten anything for two days. Headache was excruciating. One-half of a grain of morphia was required each night to produce sleep, bromide of potassium making her wild and delirious. During the latter part of the day she frequently talked incoherently.

September 15. The temperature of the left Rolandic station was 5.5° higher than that of the right. The intermitting character of the respiration formed a very interesting feature of the case at this time. There was no gradual rise and fall of the respiratory movements as seen in the typical Cheyne-Stokes' respiration. There were twenty-eight shallow respirations per minute. After four or five short and rapid respirations there was an interval of rest of eight or ten seconds, then after about the same number of respirations, came another interval of rest.

During the next week, the pain in the head became more intense, and localized in the top of the head and right temple. The bowels failed to respond to various purgatives and enemata.

September 22. The muscles of the left side of the face showed but little loss of power. The pupils were of equal size. The left leg, as well as the left arm, was now entirely paralzed. The muscular strength on the right side did not fail faster than is usual in one whose general vitality is being lowered.

The conjunctiva of the right eye was congested and watery, the sight being but little impaired.

Temperature, right axilla, 97.6°; left axilla, 98.2; pulse 68; respirations 12 per minute. Respiration was still of the abdominal type. The movements of the abdomen preceded those of chest, and formed with them a see-saw like, or to-and-fro motion, the chest apparently not expanding until the end of inspiration and beginning of expiration.

September 23. Temperature of room, 82°; of right axilla, 98.9°; left axilla, 98.2°; pulse, 72; respirations 9 per minute.

*Surface Temperature of the Head.*

| RIGHT SIDE. | | | LEFT SIDE. | | |
|---|---|---|---|---|---|
| Anterior central station, 96.3°. | | | | | |
| Anterior frontal station, | | 97.9° | Anterior frontal station, | | 96.2° |
| Posterior " | " | 95.6° | Posterior " | " | 97.6° |
| Parietal | " | 97.6° | Parietal | " | 98.1° |
| Rolandic | " | 97.0° | Rolandic | " | 96.8° |
| Occipital | " | 96.9° | Occipital | " | 98.2° |

The pulse, although only 72 per minute, was very weak, but regular in time and volume. The intermitting character of the respiration was more marked on this than on the preceding day. Three or four respiratory movements were followed by an interval of rest, lasting from twelve to fifteen seconds. The number of respirations did not exceed four in succession, nor were they, at any time, less than three. The interval, or rest, was not longer than eighteen seconds, nor was it less than twelve. The respirations, which were largely abdominal, had to be counted for two minutes to obtain the average number per minute. If the movements of respiration were looked for at the beginning of an interval, none were seen during the first quarter or third of the minute, and only three or four during the second quarter or third; whereas, during the latter part of the minute, there might be eight, six, or only three respirations. Thus:

| Inter. | Respirations. | Int. | Res. | Int. | Res. | Int. | Res. | Int. | Res. | | Respirations. |
|---|---|---|---|---|---|---|---|---|---|---|---|
| | 4 | | 4 | | 4 | | 4 | | 4 | = | 20 |
| 15s. | 9s. | 15s. | 9s. | 15s. | 9s. | 15s. | 9s. | 15s. | 9s. | = | 120 sec. |

It will be seen by observing the above tabular view, that, if the respirations had been counted during one minute only, eight might represent the breathing frequency per minute; and if those paroxysms of respiration, if I may so term them, had been limited to three respiratory movements each, as they sometimes were, six, instead of ten, of the tabulated form, could have been set down as the number of respirations. This character of breathing, sometimes slightly increasing in frequency, continued until a few days before her death, when it changed to the so-called Cheyne-Stokes' respiration.

Sighing rarely occurred. The pupils were normal. The bowels had not been opened since the 11th instant, notwithstanding various purgatives and enemata had been given. Croton oil had been given by the mouth until it had produced griping pains, and an emesis of glary mucus, yet it seemed to have no effect on the bowels. The patient lived from the 11th of September to the 13th of November without having an evacuation from the bowels. During this period of two months and two days, or about nine weeks, she neither suffered from nausea, vomiting nor distension of the bowels from gas or feces. Her diet for nearly two months just preceding her death was almost exclusively of a liquid nature, she rarely taking any semi-solid food.

September 28. During the last few days the face had been turned to the right, and the head drawn toward the right shoulder. At this time she complained of great pain and soreness in the right and posterior portions of the neck. The parts were hard, swollen, and painful to the touch. An attempt, on my part, to straighten the neck greatly augmented her suffering. Pain was also complained of in the right eye and ear. The former was inflamed, and almost useless as a visual organ, and from the latter there was a constant discharge of yellow pus. The ocular inflammation involved more especially the conjunctiva and cornea. An ophthalmoscopic examination of the right eye was unsatisfactory, on account of the hazy condition of the cornea, and the uncontrollable movements of the eyes. The watch was not heard by the right ear, except on firm pressure.

October 3. She was blind in the right eye and deaf in the right ear. The discharges from the eye and ear continued.

October 13. She remained quiet during the day, but required two grains of morphia to induce sleep at night. The hearing in the left ear varied; at times she heard tolerably well with this ear, and at others she was almost deaf. No improvement in the right eye and ear. A semi-choked disk appearance was noticed in the left eye, and vision was nearly gone. The left eye showed no evidence of external inflammation. No discharge was noticed from the left ear.

Novembr 11, 5 P. M. Temperature, right axilla 95.5° ; left axilla 98.2° ; pulse 104 ; respiration, 28 per minute.

The right side of the body felt quite cool. The breathing had changed from an intermitting to an irregular character, and constituted what is known as the Cheyne-Stokes' inspiration, except that there was no lull, during which the hand could not detect feeble movements of the thoracic wall. The respiratory movements gradually became shorter, until they were almost imperceptible, then they gradually lengthened until a full inspiration was taken with considerable effort, followed by a blowing noise. Every paroxysm, or rise and fall of the respiratory acts, included about eighteen respirations. She neither saw nor heard, and was no longer able to speak or swallow. The saliva was wiped from the mouth by the attendant, or she made an awkward and ineffectual effort to do it herself, showing that she was probably not entirely unconscious. Her urine was passed involuntarily. No morphia had been required during the last few days. The neck remained in about the same condition that it was on September 23. The muscles on the left side of the face were paralyzed, the mouth being drawn well over to the right side.

She died, exhausted, November 13, 1881, at 12:30 P. M.

*Post-mortem* examination was made three hours after death, by Drs. Mills, Bissey, and myself.

*Brain.*—The venous sinuses and arteries were carefully examined, but were found to present nothing abnormal. No lesion of the membranes was discovered. The pia mater was unusually transparent (almost bloodless). The optic nerves were normal in appearance, and no perceptible lesion was detected in the other cranial nerves. No effusion. Entire surface of brain examined through the transparent pia mater before its removal, and no pathological condition was discoverable. On stripping the pia mater from the right hemisphere, the external surface of the convolutions was still found to be intact. Before completing the process of removal of the pia mater from the right hemisphere, a large quantity of a dirty grayish-white liquid suddenly escaped through a small opening at the bottom of a secondary fissure just in front of the upper extremity of the fissure of Rolando.

The right lateral ventricle was laid open by an incision through the lateral border of the corpus callosum. The surfaces of the intra-ventricular ganglia were healthy in appearance. The floors and roofs of the cornua showed no lesion. A longitudinal incision was made through the middle of the head of the caudate nucleus and the optic thalamus, deep enough to pass through these bodies and the lenticular body, just to the inner margin of the insular and sphenoidal cortical substance (gray matter). This incision exposed a small isolated abscess about one inch in length, three-quarters in depth, and one-third of an inch in width, involving the following parts from before backwards; the anterior two-fifths of the lenticular nucleus, and the adjoining portions of the external capsule and claustrum. The caudate nucleus, optic thalamus, and the entire internal capsule, were not changed in appearance. The posterior three-fifths of the lenticular nucleus, and a very narrow strip of its anterior two-fifths above the abscess, were intact. Vertico-transverse sections through the prefrontal and occipital regions showed no lesions. Further transverse sections revealed a nodulated mass and large abscess of the centrum ovale of the postero-frontal and parietal lobes.

The nodule was about the size of a large hickory nut. It involved the white matter and the under portion of gray matter at a point corresponding externally to the superior parietal lobule or convolution, a little anterior to the middle of the inter-parietal fissure. Its anterior extremity just grazed the middle portion of the gray matter of the ascending parietal convolution. The nodule was apparently firm. The abscess extended on all sides of the irregular body, and the exact limits of the former were determined by transverse sections to be as follows: Its anterior extremity reached a point in the centrum ovale corresponding to a plane, just in front of the caudate and lenticular bodies, externally through the anterior halves of the first, second, and third frontal convolutions. Its posterior extremity reached a point in the centrum ovale of the parietal, near the plane of the parieto-occipital fissure. The abscess in its anterior half involved the white matter only, but close up to the gray substance. Its posterior portion involved the white matter, also the inner part of the gray substance of the adjoining convolutions.

The posterior one-fourth of the abscess involved the gray matter to the greatest extent, and approached near the surface at the most posterior part. It had, therefore, destroyed the centrum ovale directly in relation with, *a*, the posterior parts of the first and second frontal convolutions; *b*, the middle regions of the ascending frontal and ascending parietal convolutions; *c*, the middle and lower portions of the superior parietal convolutions; *d*, the upper portion of the inferior parietal convolution. The gray matter destroyed was chiefly in the anterior parietal convolution and the superior parietal lobule. The entire length of the abscess was between four and five inches.

The left hemisphere showed no pathological lesions.

*Thorax.*—Heart and large vessels were normal in appearance. The apex of the left lung contained four cancerous nodules, varying in size from that of a lima bean to a horse-chestnut. The apex of the right lung was the seat of a nodule about the size of a hickory-nut. The middle and lower portions of the lungs were anæmic. The pleuræ were normal, except at the apices of the lungs, where slight adhesions were formed over the nodular growths. Slight effusion in the left pleural cavity.

*Abdomen.*—The uterus, ovaries, and ligaments of the womb were the seat of fourteen cancerous nodules, most of which were not larger than a hickory-nut. The liver, spleen, kidneys, and pancreas were anæmic, but free from cancerous growths. The stomach was empty, pale, and contracted, but no induration of its walls was discovered. The upper and lower bowels contained a slight amount of fecal matter scattered throughout their entire extent. The contents of the bowels were soft. No evidence of intestinal obstruction could be detected. The rectum was empty.

*Microscopical Appearances.*—The nodule in the brain was composed of blood-vessels, brain substance, extravasated blood, and blood-pigment. No cells of a suspicious character were seen.

*Remarks.*—The abscesses cannot be attributed to the presence of a cerebral tumor. The history of the removal of a malignant growth from the left breast one year before, naturally suggested to me the probability of a similar one in the brain. No local interference with the venous or arterial circulation of the brain was detected at the autopsy. As causative agents, apparently nothing beyond a profound lowering of the vital forces and the blood infection attendant upon the presence in the system of several cancerous masses, were found. If in these lies a sufficient cause, I am at a loss to know why abscess of the brain is so infrequently found in connection with cancer of other portions of the body, or why it is not more generally associated with general anæmia.

Remittent respiration is referred to by Hughlings Jackson (Reynold's *System of Medicine*), although he does not give it that name, and Cheyne-Stokes' respiration by nearly every recent author on nervous diseases, but I find no allusion to the peculiar intermittent variety found in this case, in which three or four respiratory movements of equal length and depth were followed by distinct intermissions, varying in length from fifteen to eighteen seconds.

Just before the patient's death, when the respiration approached the Cheyne-Stokes character, there was no period or interval of several seconds duration, during which the respiration intermitted, but the movements were continued regularly, increasing or lessening as the efforts at respiration reached the maximum or minimum.

One or more cases with respirations differing from the Cheyne-Stokes' similarly to this may be found described in a joint paper presented at the Northern Medical Society of Philadelphia, by Drs. Mills and Ott (*Phila. Med. and Surg. Reporter*, July 2, 1881.

The abdominal respiration noted in the clinical history may be imitated by any one in health by first voluntarily expanding the abdomen, then allowing the chest to be inflated subsequently, the expiratory movements taking place in the order of time, in which the inspiratory were effected. It differs from the form of abdominal breathing referred to by Hughlings Jackson in connection with cerebral hæmorrhage. It is a grave omen, and is most commonly found in cases of great prostration, as in typhoid fever and shock, or it is associated with certain brain diseases attended by a similar condition of the vital forces.

The inflammatory changes that occurred in the right eye and ear, with the subsequent loss of vision and hearing in these organs, cannot be accounted for by any appreciable lesion in the pons, medulla, or at the base of the brain adjacent to the optic nerve. The well-known experiments of Waller, Bernard, and Brown-Séquard, on the effects of stimulation and paralysis of the cervical ganglia of the sympathetic nerve aid us in attributing the trophic changes found in the eye to the local trouble present in the right side of the neck, which preceded the affection of the organs of the special senses. I regret that we failed to examine the indurated tissues of the neck, and to ascertain the condition of the sympathetic nerve and ganglia in this region.

The general and cerebral thermometric records of this case present some points of interest.

*General Thermometry.*—According to the physiological experiments of Hitzig, Eulenberg and Landois (quoted by Mills, *Phila. Med. Times* for January 18, 1879), the heat-controlling centers are situated in the cerebral cortex of the motor regions, around the fissures of Rolando, especially in the ascending convolutions. Usually, but not constantly, destruction of this area gave rise to increased, and irritation produced lessened temperature in the extremities of the opposite side of the body. In the present instance, the large abscess involved to some extent the supposed thermic area of the right side. Comparative observations of each axillary temperature were made on seven different occasions, and were as follows:

| | Right Axilla. | Left Axilla. |
|---|---|---|
| Sept. 8 | | 99° |
| " 12 | 96° | 99.4° |
| " 13 | 97.9° | 98.4° |
| " 14 | 97.9° | 98.2° |
| " 19 | 97° | 98° |
| " 22 | 97.6 | 98.2° |
| " 23 | 98.9° | 98.2° |
| Nov. 11 | 95.5° | 98.2° |

The temperature of the right or non-paralyzed side of the body only once exceeded that of the left, when the thermometer registered 7° higher in the right than in the left axilla. The temperature of the left armpit ranged from 5° to 3° higher than that of the right. During the first shock to the system produced by the abscess, and just before death, when paralysis was most complete, the difference between the two sides of the body heat was greatest, being 3.4° at one time, and about 3° at another.

*Cerebral Thermometry.*—I shall only refer to the extended observations of Broca, Gray, Maragliano and Sepelli, on cerebral thermometry in health and disease. Their conclusions are in harmony with each other, and their utility has been demonstrated by observations made on at least two cases of tumor of the brain, one by Dr. Charles K. Mills (*Phila. Med. Times*, January 18, 1879), and the other by Dr. Landon Carter Gray (Hamilton's work on Nervous Diseases, 2d ed., p. 22), who was the first in this country to investigate the surface temperature of the head in health. Dr. Gray found the average normal temperatures of the stations on the left side of the head to be for the frontal, .65° ; for the parietal, .85° ; for the occipital, .72°, higher than those of the corresponding stations on the right side. He gave as the average normal temperature of the right frontal station, 93.71 ; of the left, 94.36° ; of the right parietal, 93.59° ; of the left, 94.44° ; of the right occipital, 91.94°, of the left, 92.66°. Variations of more than 1.5° he considered suspicious of disease at that point, and of more than 2° strong evidence of a pathological condition.

Comparing the results of the surface temperatures of the head made on the case reported at length in this paper with those reported by Gray and others, we shall find several points of difference. The temperatures were from 2° to 4° higher than those obtained on the heads of healthy subjects, although the axillary temperatures of this case, taken at the same time, were normal or subnormal. The temperatures at the several stations on the left side of the head exceeded those on the right by about .5°, which is nearly in accord with the normal difference. The temperature at the left Rolandic station was .2° lower than that of the right on one occasion only. With this exception, the temperatures on the left side were the highest. On September 23, when paralysis of the left side of the body was complete, the surface heat of the left posterior frontal station was 2°, and at the left parietal 1.5° greater than at the corresponding stations on the right side. Before complete paralysis had taken place in the left leg, there was nearly an equal increase of heat at the frontal and posterior stations; later, when the leg was powerless, the temperatures of the posterior stations exceeded their normal more than did those of the anterior. In the observations on cerebral thermometry in connection with brain tumors made by Drs. Mills and Gray, the stations nearest the growths, both of which happened to be on the right side, gave the highest average increase of temperature.

The area to which the occluded vessels in embolic hemiplegia are distributed is said to have a subnormal temperature.

I am not aware that the results of any observations of the surface temperature of the head made on persons suffering from abscess of the brain have been reported. No valuable conclusions can be drawn from observations made on a single case; especially is this so when the surface temperatures of the head are not more frequently taken than in the history of the one just reported. In health the head temperatures are from 4° to 6° lower than that of the axilla, being lowest posteriorly. In the present case, with a normal or subnormal axillary temperature, the head temperatures were elevated from 4° to 6° above their normal, the occipital, in one instance, being the highest, equalling the axillary, and exceeding its own in health 6°.

Dr. Allan McLane Hamilton, of New York, has reported the results of his observations on a case of meningitis, in which the occipital temperatures exceeded those of the other regions of the head. (Ibid.)

It is doubtful whether the local temperatures of the head in abscess of the brain will aid us in locating the disease to any particular region, but if the results of the observations already made on cerebral thermometry in brain tumor be sustained by future investigations, the surface thermometer may help us in differentiating abscess from tumor of the brain.

In a case of brain trouble, in which the diagnosis is between tumor and abscess, a limited surface area of one side (especially the right) of the head, with a temperature considerably elevated above the heat of the surrounding parts, would point to tumor; whereas, an elevation of the entire cerebral surface temperature would indicate abscess.

The direction of the destructive lesion in the brain, if we may judge by the progress of the paralysis, was from before backward, showing that the nodule occupied the region last involved, instead of the first, as would have been the case if it had been caused by an embolus, and had in its turn given rise to the abscess.

CASE II. Abscess of the brain following chronic meningitis; sudden death.

On the notes of Dr. Dennis the history of the following case is based:—

Mr. G. S., æt. 56, German, shoemaker, served in the German army, and, when a young man, led an irregular life. He presented no marks of syphilis, and no history of his having suffered from that disease could be ascertained. He had always been a moderate drinker of beer, but never indulged in the stronger alcoholic liquors.

He was a large, heavily built man, and, with the exception of occasional headaches, had enjoyed good health until the latter part of the year 1880, when the loss of his wife, followed by other domestic unhappiness, seemed sufficient to disarrange an already ill-arranged nervous system. His temper had been irritable for many years, but now he became subject to frequent outbursts of passion, during which he would vent his spleen on any one who chanced to be near him. His memory became very treacherous, often getting confused in his business, and even at times being unable to remember the day of the week. His headache became constant and more severe.

During the spring and early part of the summer of 1881, he had what he called "rheumatic attacks," during which, while attending to his usual duties, he would be seized suddenly by a sharp pain in the course of the left sciatic nerve, followed by immediate paralysis of the left arm and leg, compelling him to sit down to save himself from falling. During the attacks he did not lose consciousness. The paralysis never lasted longer than a few minutes, power of motion returning, and seeming to be as good as before the shock. The frequency of the hemiplegic seizures, two or three attacks often occurring weekly, depended upon causes giving rise to outbursts of passion. Sudden changes of weather were apparently associated with the nervous disturbance.

His symptoms remained unchanged until the latter part of October, when, in a fit of anger, he fell to the floor, frothed at the mouth, and became convulsed. No paralysis followed the convulsion, but the headache steadily increased, compelling him to take to his bed. As on this, so on every succeeding day during his entire illness, he remained in bed only a portion of the day.

The head-pain, now constant, was so severe that it prevented continuous sleep, and from short naps, which he would occasionally get, he would be awakened suddenly by violent exacerbations of pain.

He was attended by homœopathic physicians until November 12th, two weeks before his death, when Dr. Dennis was requested to take charge of the case. His temperature at this time was 99.5° ; pulse 70 per minute, full and strong ; respirations natural ; appetite capricious ; tongue coated, stomach intolerant, and bowels obstinately constipated. Urine scanty, high-colored, and free from albumen, was voided voluntarily until a short time before his death, when it was occasionally passed involuntarily. The skin was slightly jaundiced. He complained of occasional chills, and was very sensitive to changes in the atmosphere. Nocturnal delirium soon begun. He was very restless, saw strange things and people in his room, and would occasionally rise during the middle of the night and awake the whole family, telling them that it was time to get up for the day. At times his mind seemed to be a blank, he could not tell the day of the week, and would frequently be unable to distinguish day from night. On other occasions his mind was clear, but during these lucid intervals he could converse intelligently and connectedly for a few minutes only, thought suddenly ceasing to be generated, when a vacant stare would be his only reply to questions which a few minutes before he had answered promptly and correctly. Impressions were persistently and obstinately adhered to, and any attempt at persuasion to the contrary was not calmly received. His headache, principally frontal and right-sided, was now sharp and lancinating, requiring hypodermic injections of morphia and atrophia to relieve it. The orbital and other branches of the fifth nerve were very sensitive to pressure. His temperature during

Dr. Dennis's attendance, though usually above the normal, reached 100° Fah. only on two or three occasions. The muscles of the left arm and leg were decidedly weaker than those of the right, yet sufficiently strong in the leg to support his weight for a short time. Turning in bed was difficult. When the left arm, unaided, was held at right angles with the body a decidedly muscular twitching began. Occasional muscular twitchings took place when the arm was passive.

He thus continued, gradually getting worse, until November 25th, about five weeks after his giving up work, when Dr. Dennis requested me to see him in consultation.

When I first entered the room he seemed bright, and conversed intelligently. He had not talked long, however, before he began to hesitate in his answers, appearing to have difficulty in understanding me, and being more puzzled in framing answers to my questions. Soon he was unable to form more than one or two words of a sentence, and finally uttered a meaningless "Yes" or "No," or stared vacantly when spoken to. Pupils were small and equal in size, brows were contracted, and he complained of great pain in his head. The eyes were very sensitive to light, making it extremely difficult to make an accurate or satisfactory ophthalmoscopic examination. The acuteness of vision and the condition of the other special senses could not be ascertained. The retinæ were found to be slightly hyperæmic, and the optic disks somewhat swollen. No muscle or groups of muscle were paralyzed, but those of the left arm and leg were weak, and an attempt at co-ordination produced a muscular tremor. The left side of the face was apparently less expressive than the right. The heart, lungs, liver, and kidneys showed no evidence of disease. Temperature was 100°; pulse, 68, and full; respirations 16 per minute.

An opinion was given that the man was suffering from chronic meningitis of months' duration, and that a recent complication of softening or abscess of the brain had taken place.

As Dr. Dennis had enjoined rest in bed, and was doing everything necessary for the comfort and welfare of his patient, no change in treatment was made.

The next day Mr. S. was feeling so comfortable that he got up, dressed himself, and without assistance went down stairs from the third to the first floor to dinner. After eating he was helped to his third-story room. He expressed himself as feeling tired and sleepy, and lay down to rest. He was left alone about one hour, when one of his daughters looking in his room found him on the floor, where he had fallen in getting out of bed. The left side was now found to be entirely paralyzed. He was placed in bed and left alone. Shortly after this, when looked after, he was found asleep and snoring loudly. As his repose was supposed to be a natural one, he was not immediately disturbed. About 6 P. M. the daughter, thinking it strange that nothing was heard from her father, went into his room and endeavored to arouse him, but to her surprise found him dead.

Post-mortem examination thirty-six hours after death.

*Brain.*—The dura mater, over the entire extent of its bony attachment, was inflamed and firmly adherent to its osseous envelope. At the base of the brain the external membrane was so closely attached that it could be separated from the bone only in piecemeal by the scalpel. The cranial nerves were apparently uninvolved in the diseased process. No abnormal quantities of fluid were found in the subarachnoid space. The blood-vessels of the pia mater were engorged, and the membrane was slightly adherent to the brain substance. The entire external surface of the brain appeared nearly normal, presenting no gross lesions of softening. Superficial portions of convolutions in the neighborhood of the greatest amount of inflammation were examined under the microscope, and found to be in nearly a normal condition.

An abscess was found on the right side, involving chiefly the centrum ovale of the pre-frontal region, but also to some extent, in its middle part, the inner layers of gray matter of the first, second, and third frontal convolutions. On attempting to localize exactly the posterior limits of the abscess, it was found that a vertico-transverse section through the anterior lobe made just in front of the pre-central fissure externally, and striking the anterior extremities of the ganglia internally, just grazed its posterior limits. The abscess extended forward to within one inch of the anterior extremity of the lobe. Very few, if any, of the white fibers from the ascending frontal convolutions were involved. At the posterior and interior boundaries of the abscess a bloody nodulated mass two-thirds of an inch in its greatest dimensions was found. Other portions of the brain were apparently healthy. No emboli or thrombi were found.

The abscess did not communicate with the lateral ventricle, nor open upon the external surface of the brain to account for sudden death.

Microscopic examination of the nodulated mass and adjacent parts showed no new cell formation tissue; the nodule and the parts around exhibited nothing besides disintegrating brain tissue with its blood-vessels, and a few blood corpuscles.

*Remarks.*—We cannot boast of our knowledge of the functions of the antero-frontal lobes, yet pathology, and later, physiology, have from time to time thrown some light on this obscure subject. The "crow-bar case" of Bigelow, the bullet wound related by Trousseau (quoted by Ferrier), the other cases mentioned, analyzed by Charcot and Pitres (reviewed by Ferrier, and quoted by Mills, *Phila. Med. Times*, Jan. 18, 1879), the tumor of the right antero-frontal lobe presented to the Pathological Society of Philadelphia, by Dr. C. K. Mills (*Transactions of Philadelphia Pathological Society*, vol. ix. page 106), and the lesion just described in connection with case second of this paper, in all of which the antero-frontal lobe was destroyed to a greater or less extent without any paralysis attributable to the leison in this region, lead us to accept the conclusion of Charcot and Pitres, that motor disturbances of the slightest character do not necessarily follow lesions in the antero-frontal regions.

For the light physiology has shed upon the functions of this region of the brain, I quote from Dr. Mills's article on tumor of the brain (*Phila Med. Times*, Jan. 18, 1879): "Ferrier, *Functions of the Brain*," p. 230, in his experiments on the antro-frontal regions of the brain of the monkey, generally obtained negative results. Electrical irritation usually caused no special manifestations. Removal or destruction was not followed by any definite physiological results. The animals operated on, however, while not actually deprived of intelligence, had apparently lost the faculty of attentive and intelligent observations. When considering the hemispheres physiologically, Ferrier argues also that the power of fixing the attention and concentrating consciousness depends on inhibition. He shows that if the centers of inhibition, and thereby the faculty of attention, are weak, or present impulses unusually strong, volition becomes impulsive rather than deliberate."

It will be remembered that the patient in case second, where the leison was in the antero-frontal lobe, presented symptoms in accord mainly with the physiological experiments of Ferrier. The man was emotional and passionate, and his power of attention was weak and of short duration. He seemed at times to lose will power. Only temporary paralysis was observed, and this always after some fatigue, which may have induced some restraining influence from other portions of the brain, or what is probably more correct, as some of the fibers connecting the motor region of the brain with the muscles of the arm and leg were destroyed, the remaining healthy fibers became exhausted from overwork.

Among the other interesting features of the second case are, the sudden death and the patient's ability at times to walk well, although at others the paralysis was so extensive that he was not able to stand.

That he was able to walk at all was apparently due to the fact, sustained by the post-mortem examination, that not all of the fibers through which motor impulses passed from the brain to the muscles of the leg were involved in the destructive process. When he was resting, a few fibers, for a short time, could do the work of a greater number.

If this view be correct, it will enable us to explain the cause of sudden death, although there was neither hæmorrhagic apoplexy, nor bursting of the abscess into the ventricles, or on the surface of the brain. The unusual strain that he put upon his crippled brain a few hours before his death, in descending and ascending two flights of stairs, and in sitting up to eat his dinner, so exhausted his nerve centers that its effects came like a shock from a severe cerebral hæmorrhage. The occurrence of the hemiplegic seizures may be accounted for, probably, by the sudden overloading of the congested vessels of the brain during his fits of anger.

CASE III.—Abscess of the brain; gangrenous inflammation of the right sylvian artery, and occlusions of some of its branches; facial erysipelas; pneumonia in the left lung.

J. R., male, aged about 40, laborer in a sugar refinery, had been sick and delirious for several days, was brought into St. Mary's Hospital during the past winter, in an unconscious condition. His previous history could not be ascertained. His respirations were rapid and abdominal; pulse small, weak, and irregular; temperature not taken. No urine was obtained for examination. His face was swollen and of a dark erysipelatous hue. The swelling around the eyes was too great to admit of an examination of the pupils. The facial swelling and comatose condition prevented the detection of local palsies, if such existed. He did not regain consciousness, but died about six hours after his admission.

*Autopsy eight hours after death.*—The abdominal cavity was not opened. The entire left lung was in the second stage of pneumonia; the right lung and the heart were in nearly normal condition. Only slight quantities of serous fluid were found in the pericardium and right pleural sac; considerable fibro-serous effusion was found in the left pleura.

*Brain.*—The dura matter was slightly congested, but there were no evidences of decided inflammation in it. The pia matter covering the convex surfaces of both hemispheres, and that beneath the right was opaque. Its blood-vessels were highly engorged, and deposits of lymph and pus were found. At and just posterior to the right Sylvian fissure there was considerable quantity of pus, and sufficient lymph to so mask the structures at this point as to make them look like one mass of inflammatory products. The trunk of the Sylvian artery was almost black, its caliber lessened, but apparently not entirely obliterated. Some of its secondary branches, especially the lower ones from the parieto-sphenoidal and sphenoidal arteries were occluded and seemingly gangrenous. After carefully removing the pia matter, the convulsions and brain substance of the left hemisphere appeared nearly normal. The under and external surfaces of the right temporo-sphenoidal lobe from its anterior tip to its junction with the occipito-parietal lobules were softened to the depth of about an eighth of an inch. A superficial abscess or a mass of softened brain substance, about one inch in all its dimensions, oc cupied the anterior extremities of the first, second and third temporo-sphenoidal lobules. The contents of the abscess were, in the center, a watery fluid, surrounded by broken down brain substance and blood constituents, and on the outside, a thick layer of pus and other exudative matter.

The cranial nerves were but little encroached upon by the inflammatory deposits. Nothing special was noticed in the sinuses of the brain besides their blackened and engorged appearance. The ganglia were apparently normal.

The patient was not seen by me during life, and he was under the observation of Dr. Strittmatter, the resident physician of the Hospital, only six hours before his death. The circumstance of his death was investigated by the coroner, which necessitated great hurry in examining the brain and its membranes and sinuses. The consideration of the cause of the abscess and meningitis, and the nature of the erysipelas, I shall leave for those who may take part in the discussion.

[After the reading of the above paper, Dr. Charles K. Mills said:

A careful examination of the cerebral sinuses might have thrown some additional light upon the third case. It was of great importance in all cases of abscess of the brain to investigate the condition of the sinuses of the dura mater. Abscess and thrombosis were not infrequently associated, and sometimes held to each other a causative relation. In the account of the condition of the patient (Case III.) it was stated that his face was swollen, and of a dark erysipelatous hue, the swelling around the eyes being too great to admit of an examination of the pupils or of the interior of the eyes. The erysipelatous appearance may have been the psuedo-erysipelas which accompanies some forms of cerebral thrombosis. The opthalmic veins end in the cavernous sinuses. These veins have also a communication with the veins of the face. The facial vein, beginning on the anterior portion of the head, descends about the middle of the forehead, as the frontal vein, to the root of the nose, and is then continued downwards around the angle of the eye, under the name of the angular vein. This angular vein communicates with the ophthalmic vein. Hence it will be seen how swelling of those veins outside of the skull, which inosculate with the sinuses within, may become the most striking and positive signs of obstruction of the sinuses. Œdema of the eyelids and face, and congestion of the eyes, mean venous stasis in the parts drained by the ophthalmic veins. These phenomena were present in Dr. Eskridge's third case.

These three cases of abscess of the brain, well studied and described, constitute a valuable addition to the literature of intracranial diseases.—*Phil. Medical Times.*

